# Sleep and the Pharmacotherapy of Alcohol Use Disorder: Unfortunate Bedfellows. A Systematic Review With Meta-Analysis

**DOI:** 10.3389/fphar.2019.01164

**Published:** 2019-10-17

**Authors:** Francesca Panin, Alessandra T. Peana

**Affiliations:** ^1^Faculty of Health, Education, Medicine and Social Care, Anglia Ruskin University, Cambridge, United Kingdom; ^2^Department of Chemistry and Pharmacy, University of Sassari, Sassari, Italy

**Keywords:** alcohol use disorders (AUD), sleep disorder, insomnia, pharmacotherapy, disulfiram, acamprosate, naltrexone, nalmefene

## Abstract

**Background:** Sleep disorders are commonly associated with acute and chronic use of alcohol and with abstinence. To date, there are four approved drugs to treat alcohol use disorder (AUD): disulfiram, acamprosate, naltrexone, and nalmefene. These AUD therapies reduce the craving and risk of relapse into heavy drinking, but little is known about their effect on sleep. As recent evidences indicate a crucial role of sleep disorders in AUD, claiming that sleep problems may trigger alcohol abuse and relapses, it is fundamental to clarify the impact of those drugs on the sleep quality of AUD patients. This systematic review aims to answer the question: how does the pharmacotherapy for AUD affect sleep?

**Methods:** We searched PubMed, Embase, CINAHL Plus, Cochrane, and Scopus using sleep- and AUD pharmacotherapy-related keywords. The articles included were appraised using the CASP checklists, and the risk of bias was assessed following the Cochrane risk-of-bias assessment tool. Finally, we pooled sleep outcomes in a meta-analysis to measure the overall effect.

**Results and Conclusion:** We included 26 studies: only three studies focused on sleep as a main outcome, two with polysomnography (objective measurement), and one with subjective self-reported sleep, while all the other studies reported sleep problems among the adverse effects (subjective report). The only study available on disulfiram showed reduced REM sleep. Acamprosate showed no/little effect on self-reported sleep but improved sleep continuity and architecture measured by polysomnography. The two opioidergic drugs naltrexone and nalmefene had mainly detrimental effect on sleep, giving increased insomnia and/or somnolence compared with placebo, although not always significant. The meta-analysis confirmed significantly increased somnolence and insomnia in the naltrexone group, compared with the placebo. Overall, the currently available evidences show more sleep problems with the opioidergic drugs (especially naltrexone), while acamprosate seems to be well tolerated or even beneficial. Acamprosate might be a more suitable choice when patients with AUD report sleep problems. Due to the paucity of information available, and with the majority of results being subjective, more research on this topic is needed to further inform the clinical practice, ideally with more objective measurements such as polysomnography.

## Introduction

Alcohol use disorder (AUD) is a chronic relapsing brain disease characterized by compulsive alcohol use, loss of control over alcohol intake, and a negative emotional state in absence of alcohol. When people, physically dependent on alcohol, stop drinking, they experience a very unpleasant alcohol withdrawal syndrome. The symptoms usually resolve spontaneously within a week, but more severe forms may be associated with generalized seizures, hallucinations, and delirium tremens, which can be fatal (Jesse et al., 2017).

**Figure 1:**
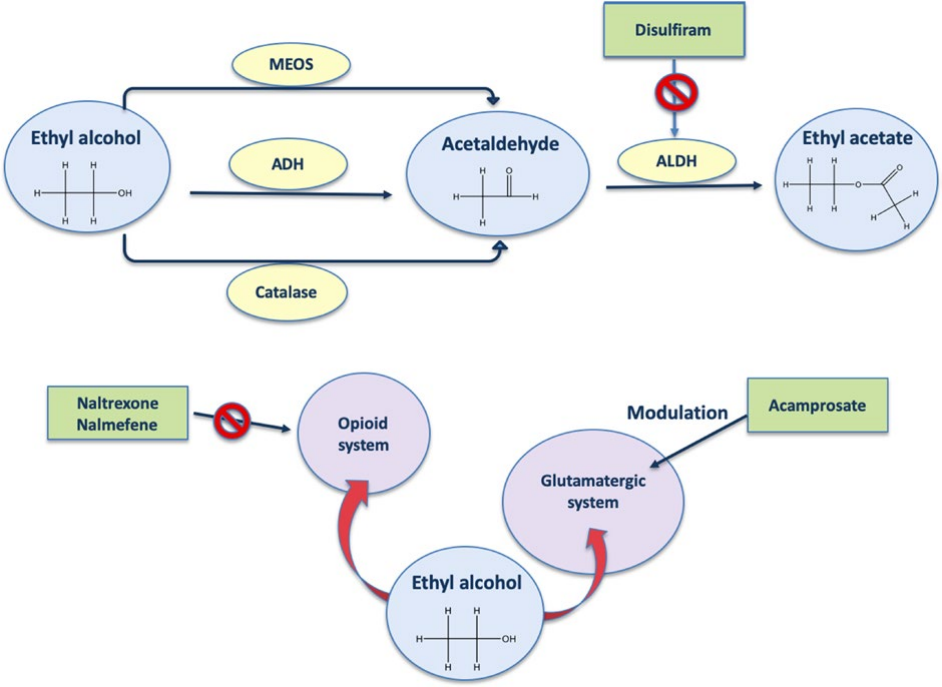
Mechanism of action of AUD drugs. ADH, alcohol dehydrogenase; ALDH, aldehyde dehydrogenase; MEOS, microsomal ethanol oxidizing system.

To date, the drugs to treat AUD are disulfiram, acamprosate, naltrexone, and nalmefene (**Figure 1**). Disulfiram, well known as antabuse, was the first medication approved by the U.S. Food and Drug Administration (FDA) to treat alcohol dependence in 1951 (Kragh, 2008). Disulfiram should only be used in patients who are highly motivated to maintain abstinence (Alozai and Sharma 2019). Its mechanism of action for maintaining alcohol abstinence is thought to be primarily psychological (Skinner et al., 2010) and based on a highly unpleasant pharmacological consequence if alcohol is consumed. In fact, disulfiram blocks the enzyme aldehyde dehydrogenase; thus, in the presence of alcohol, elevated concentrations of its metabolite acetaldehyde (**Figure 1**) will result in the unpleasant disulfiram-ethanol reaction consisting primarily of tachycardia, flushing, nausea, and vomiting (Swift, 1999; Zindel and Kranzler, 2014). Therapeutic and adverse effects are therefore closely connected: with disulfiram, alcohol becomes a health threat if the patient fails to maintain complete abstinence (Mutschler et al., 2016).

Acamprosate, approved by the FDA in 2004 (Kragh, 2008), is a drug known to be a glutamate receptor antagonist. In line with this, acamprosate is reported to reduce blood (Nam et al., 2015) and brain (Frye et al., 2016) glutamate levels. It has been suggested that inhibition of glutamate binding (**Figure 1**) occurs *via* allosteric interaction rather than direct competition (Harris et al., 2002). Acamprosate supports abstinence by normalizing the often-protracted dysregulation of NMDA-mediated glutamatergic neurotransmission that follows chronic heavy alcohol use and withdrawal (Mason and Crean, 2007). However, acamprosate might be able to reduce drinking-related parameters only in a certain percentage of alcohol-dependent patients (for review, see Boothby and Doering, 2005). Interestingly, recent evidences show that serum levels of glutamate are decreased by acamprosate only in positive responders, who also show higher baseline levels of glutamate with respect to non-responders, suggesting that the baseline levels of glutamate could be used as biomarkers of the response to acamprosate (Nam et al., 2015).

Use of opioid antagonists appears to reduce certain aspects of alcohol drinking in animals (Hyytia and Sinclair, 1993) and in humans (Reid et al., 1986). Naltrexone (oral and long-acting injectable formulations) is a non-selective opioid receptor antagonist (**Figure 1**) approved by the FDA to treat opioid dependence in 1984 (Kragh, 2008). The rationale for the use of opioid receptor antagonists to treat alcohol dependence dates back to 1980s, when preclinical studies first showed an interaction between alcohol and opioid receptors, involving an important role for the endogenous opioid system as a mediator of the reinforcing effects of alcohol (Hiller et al., 1981). Preclinical studies also showed the opioidergic regulation of alcohol and alcohol metabolites on drinking behavior (Lucchi et al., 1982; Myers et al., 1986; Reid et al., 1986; Peana et al., 2011), supporting the potential utility of opioid antagonists to treat alcohol dependence. Naltrexone decreases the probability of a return to any drinking and binge-drinking risk (Kranzler and Soyka, 2018). A recent meta-analysis of clinical trials on the effects of the naltrexone on alcohol drinking found overall significant efficacy, but in several studies, the effects are only minor and/or variable (Maisel et al., 2013).

Nalmefene (daily and/or *as needed*) is an opioid system modulator (**Figure 1**) with antagonist activity on the µ and δ opioid receptors as well as a partial agonist activity at the κ receptor (Nutt, 2014). This drug is the first and still the only pharmacological compound registered for reduced drinking in patients with alcohol dependence by the European Medicines Agency (EMA, 2010). van den Brink et al. (2013) observed significant diminutions of alcohol intake with nalmefene *versus* placebo, and although this drug induces many side effects, it is overall well tolerated (van den Brink et al., 2015).

Generally, these drugs for AUD (disulfiram, acamprosate, naltrexone, and nalmefene) have been largely inadequate at preventing relapse at a population level, and this may be because they only target certain aspects of AUD (Sanchez-Alavez et al., 2019). In fact, the complexity of AUD, especially from a neurobiological evaluation, makes pharmacologic treatments particularly difficult, considering the high co-morbidity with mood-related disorders (psychotic, bipolar, depressive, anxiety, sexual dysfunction, obsessive-compulsive, delirium, neurocognitive) and, in particular, with sleep disorders.

**Figure 2:**
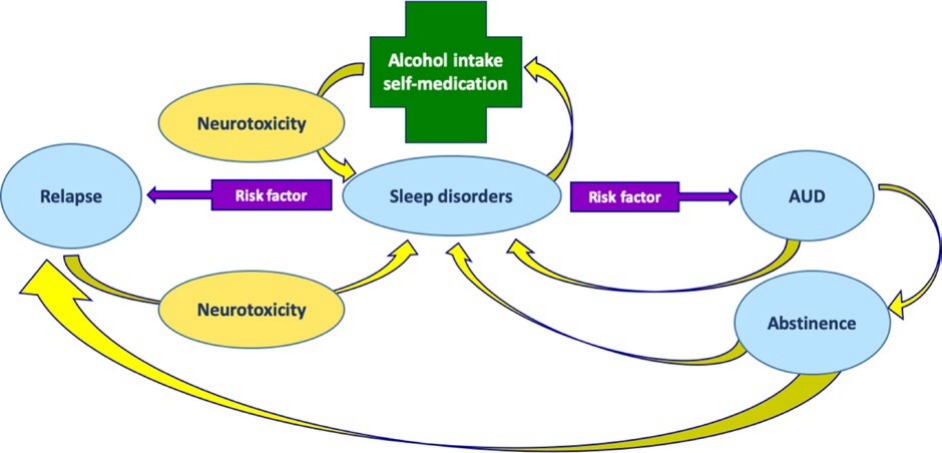
Relationships between AUD and sleep disorders. Sleep disorders may induce alcohol intake for self-medication. In turn, alcohol intake, through its neurotoxic effects, may induce sleep disorders. Sleep disorders is likewise a risk factor for AUD. Treatment of AUD can lead to abstinence, but at the same time, sleep disorders may persist even during abstinence. Sleep disorders, in turn, are risk factors for alcohol relapse. At the same time, relapse contributes to alcohol neurotoxicity and persistent sleep disorders.

Sleep problems are commonly associated with AUD (**Figure 2**); nonetheless, these symptoms are frequently ignored (Brooks and Wallen, 2014). The association between alcohol use and sleep disorders seems to be bidirectional (Chakravorty et al., 2016), as shown in **Figure 2**. Acute excess of alcohol intake induces sleep; therefore, alcohol is often used by individuals as a sleep-promoting agent to self-medicate insomnia (sleep disorder in which the quantity or quality of sleep is less than desired; **Table 2**). However, after chronic intake, the hypnotic effect of alcohol decreases (Vitiello, 1997). Clinical and preclinical studies have shown that chronic alcohol intake usually interferes with both sleep architecture (which is the basic structural organization of sleep as it shifts into stages that include REM and NREM; **Table 2**) as well as continuity (amount and distribution of sleep *versus* wakefulness) (Crum et al., 2004; Thakkar et al., 2015). On the other hand, insomnia is associated with daytime consequences that include mood and cognitive alterations (for example, reduced ability to concentrate) that might have an impact on the quality of life (Buysse, 2008). Worsening of sleep quality in AUD patients may increase the drive to use alcohol to self-medicate sleep problems, which, in turn, would further impair sleep in a dangerous vicious circle (**Figure 2**). It is therefore suggested that sleep problems increase the risk for developing AUD (Teplin et al., 2006). Insomnia occurs in 36–72% of alcoholic patients and may last for weeks to months during acute abstinence from alcohol (Brower, 2003). On the contrary, long-term abstinence from alcohol use can reverse some sleep problems (Brower et al., 2001), but long-term abstinence is difficult to achieve, and insomnia during the initial phase of abstinence might increase the risk of relapse (Gottheil et al., 1979; Brower, 2003; Arnedt et al., 2007; Chakravorty et al., 2016) as shown in **Figure 2**. Finally, insomnia can persist even after several years of abstinence, with more fragmented sleep (i.e., sleep that is interrupted by repetitive awakenings) and delayed sleep onset (Brower, 2001; Escobar-Cordoba et al., 2009).

A growing body of literature highlights the importance of addressing sleep problems to support the treatment of AUD, especially during acute withdrawal. Nevertheless, sleep problems in AUD still remain often untreated, mainly for concerns about the safety of additional hypnotic drugs that can potentially induce dependence and can interfere with alcohol (Hajak et al., 2003; Ashton, 2005; Foulds et al., 2018). In this framework, it appears particularly important to review the effects of the pharmacotherapy for AUD on sleep in order to elucidate how the drugs used to treat AUD can improve (or worsen) sleep problems during abstinence. The choice of the pharmacotherapy for AUD should be informed by evidence on the effects of those drugs on sleep. Choosing a drug that has no detrimental effect or could even improve sleep during abstinence from alcohol may play an important role in the recovery process. Furthermore, improving the quality of sleep may as well support other psychosocial interventions such as cognitive behavioral therapy for a higher therapeutic success. This systematic review with meta-analysis aims to determine how the pharmacotherapy for AUD impacts the quality of sleep.

## Methods

### Overview

**Figure 3:**
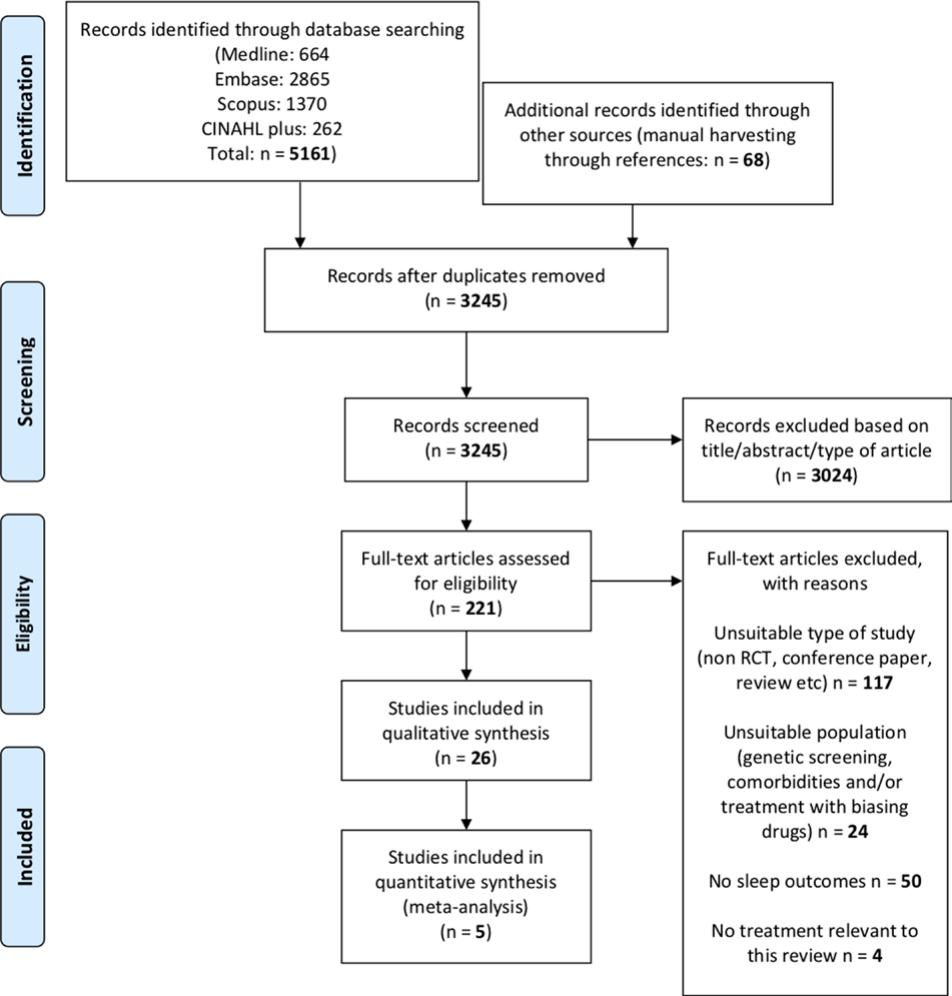
Study flow diagram of selected studies (PRISMA).

The research question was: how does the pharmacotherapy for AUD (disulfiram, acamprosate, naltrexone, and nalmefene) affect sleep? To answer this question, we followed the preferred reporting items statement for reporting on systematic reviews and meta-analyses (PRISMA; Moher et al., 2009), as shown in **Figure 3**.

According to the Cochrane handbook recommendations (Higgins and Green, 2011), the population (P), intervention (I), comparison (C), outcome (O), and study design (S) (PICOS model) was used as follows: population, healthy people with diagnosis of AUD; intervention, pharmacotherapy for AUD (disulfiram, acamprosate, naltrexone, and nalmefene); comparison, placebo/other active drug; outcome, sleep (either as main outcome or measured as an adverse effect); and study design, randomized controlled trial (RCT).

### Search Strategy

PubMed, Embase, CINAHL Plus, and Scopus were searched. Cochrane was additionally searched to exclude the existence of other systematic reviews or protocols on this topic and to use related systematic reviews for manual harvesting of additional manuscripts through the references. The first database searches were conducted in December 2018 and then updated at the end of April 2019.

Boolean “AND,” “OR,” and truncation (*) operators were applied to terms related to sleep disorders, alcohol use disorders, and pharmacotherapy (**Table 1**). Note that nalmefene was not initially included in the keywords, but since the electronic database search elicited studies on nalmefene, and given that it’s a recently added drug for AUD (Shen, 2018), it was decided to include the relative results. Searches were limited to articles published in English. The reference sections of relevant reviews were also screened for any additional manuscripts not found from the electronic database searches.

**Table 1:** Key words used for the electronic databases search. The “OR” Boolean Operator was used within each column to search for synonyms, while the “AND” operator was used between the columns to search for associations of terms.

OR	AND	OR	AND	OR	AND	OR
Sleep* disorder	Alcohol*	Use	Pharmacotherap*
			Drug therap*
Sleep* deprivation		Abuse		
			Medication
			Drug treatment
Sleep* impairment				
		Consum*		
			Pharmac* treatment
Sleep* quality				
			Pharmac* intervention
	Ethanol	Addict*		
Sleep* disturbance			Acamprosate
		Dependen*	Disulfiram
Insomnia				
			Antabuse
		Withdra*		
Sleep* problems			Naltrexone
			Pharmac* therapy

*Truncation operator to search for related words.

### Eligibility Criteria

Studies were included if they (a) were RCT, as this design provides the most reliable evidence about the effects of healthcare interventions (Guyatt et al., 2008); (b) sampled individuals with AUD; and (c) included any measure of sleep, either as a main outcome or among the adverse effects. Studies were excluded if they (a) failed to report any sleep outcome; (b) were not RCT or pooled data from other RCT studies; (c) sampled individuals with specific characteristics other than AUD (e.g., individuals genetically screened, and/or with any mental/neurological comorbidity); (d) sampled individuals with concomitant treatments known to affect sleep (e.g., benzodiazepines, antidepressants, other drugs of abuse etc.); (e) focused only on drugs other than the drugs indicated for AUD (disulfiram, acamprosate, naltrexone, and nalmefene); (f) focused only on sleep without AUD; (g) were not original, peer-reviewed articles (e.g., reviews, conference papers, letters to editor, guidelines, and notes); and (h) were not on humans.

### Screening, Data Extraction and Critical Appraisal

The two reviewers (FP and ATP) independently extracted the data and appraised the quality of the final set of studies, using the Critical Appraisal (CASP UK, 2019) checklist for RCT, and any disagreements were resolved through consensus. Throughout the CASP checklist, reviewers selected “yes” (score: 2) if the procedure (e.g., randomization) was cited and clearly described, “no” (score: 0) if the procedure was not cited, and “can’t tell” (score: 1) if the procedure was only cited but not described. The total score from the CASP checklist was also reported in the data extraction (**Table 3**).

For each study, the following information was extracted: (1) treatment and design (drug, dose, posology, and duration of treatment), (2) population’s characteristics (number of subjects per group and sobriety at inclusion), (3) any additional psychological support, and (4) outcomes of interest (sleep). Results are shown in **Table 3**.

### Risk of Bias

**Figure 4:**
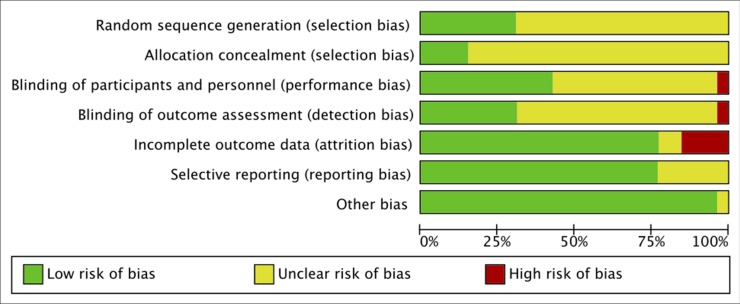
Risk of bias graph: authors’ judgments about each risk of bias presented as percentages across all included studies.

Following the Cochrane risk of bias assessment tool (Higgins and Green, 2011), reviewers determined the risk of bias for: selection, performance, detection, attrition, reporting, and other biases. Using the scores from the CASP checklist (CASP UK, 2019), the risk of bias for each domain was rated as: high (CASP: no), low (CASP: yes), or unclear (CASP: can’t tell) in the Cochrane’s Review Manager (RevMan, 2014) (see **Figure 4**). It must be considered that, since the outcome of interest of this review was not the pre-specified outcome of most studies, the relative level of risks of selective bias or other biases don’t refer to the outcomes reviewed here. In fact, in all studies where sleep measures were reported as adverse effects, it was not possible to determine at what point in the study these were assessed and how was the sleep quality before the treatment, and this represents a potential bias for this review. However, the risk of selective bias and other bias was evaluated based on the pre-specified outcomes only, following the guidelines.

### Outcomes and Meta-Analysis

The outcomes were sleep-related measures, mostly reported as adverse effects, and included insomnia, hypersomnia, sleepiness (or daytime somnolence, which is a state characterized by strong desire for sleep), lethargy, sleep difficulties not otherwise specified, and sleep measures from polysomnography (sleep onset latency etc), all described in **Table 2**.

**Table 2:** Sleep terminology.

Daytime sleepiness	As somnolence. Excessive daytime sleepiness characterized by strong desire for sleep.
Somnolence	As sleepiness.
Sleep Disorders Symptoms	Sleep disturbance. Sleeping difficulty.
Difficulty Falling asleep	Initial insomnia.
Difficulty sleeping	Difficulty falling asleep or waking up several times throughout the night.
Difficulty Staying asleep	Trouble remaining asleep.
Hypersomnia	A sleep disorder in which someone sleeps for very long periods and is always very tired during the day.
Inefficient sleep	Low sleep efficiency.
Insomnia	A sleep disorder in which the quantity or quality of sleep is less than desired, usually characterized by difficulty falling or staying asleep, or waking too early, and experiencing daytime consequences of reduced sleep.
Lethargy	Having little energy; feeling unwilling and unable to do anything.
Nightmares/hallucinations	A very upsetting or frightening dream/ an experience in which you see, hear, feel, or smell something that does not exist, usually because you are ill or have taken a drug.
Polysomnography	A technique that records brain activity, the oxygen level in the blood, heart rate and breathing, as well as eye and leg movements in order to study sleep and diagnose sleep disorders.
Rapid eye movement (REM) sleep	The phase of sleep characterized by conjugate eye movements, paralysis of other muscles, and brain activity that is most similar to wakefulness.
Non-REM (NREM) sleep	The collective sleep stages (three in total) that are not categorized as REM sleep (also called quiescent sleep).
REM sleep latency	The amount of time from the onset of sleep to the onset of REM sleep.
Sleep efficiency	The percent of time in bed spent sleeping, calculated as total sleep time divided by time in bed.
Sleep latency	The amount of time from lights out to sleep onset. Sleep onset latency.
Total REM time	The amount of REM time during sleep.
Total sleep time	The amount of sleep in one complete episode of sleep, usually reported in minutes.
Total time awake	Total period not asleep.
Wake after sleep onset	The amount of time awake after the onset of sleep and before final awakening.

The number of days of sobriety at inclusion was also considered, because we *a priori* know that alcoholism affects sleep, even after long periods of abstinence (see Brower, 2001), and therefore, we estimated that having a relatively short period of sobriety could be an important biasing factor.

For the meta-analysis, we included only studies sharing the same drug, dose, comparator, treatment length, and reporting the same sleep outcome (insomnia and sleepiness/somnolence), in order to minimize the clinical and methodological heterogeneity (Ryan, 2016).

Unfortunately, only five studies with naltrexone met the above criteria and were included in the meta-analysis. Of the five studies, four reported measures of insomnia, and four reported measures of sleepiness (or somnolence; **Table 2**). All the other studies had different design and/or outcomes, and therefore, it was not considered appropriate to pool the data together.

All the studies included in the meta-analysis reported dichotomous outcomes, which were combined using odds ratio (OR). We estimated the pooled OR of insomnia and somnolence with its 95% confidence interval (CI) using the inverse variance method with random effects meta-analysis model, assuming that the true effect size varies between studies (Borenstein et al., 2010). Statistical heterogeneity was assessed with the τ^2^, χ^2^, and the I^2^. The Z test for overall effect was also performed. The threshold of statistical significance was set at p < 0.05. All analyses were conducted with the Cochrane’s Review Manager (RevMan, 2014).

## Results

### Overall Summary of Included Studies

The search produced 5,161 articles (**Figure 3**). Application of the inclusion and exclusion criteria resulted in 26 RCT on the efficacy and/or safety of disulfiram, acamprosate, naltrexone, and nalmefene. All the relevant characteristics and outcomes of the studies are summarized in **Table 3**.

**Table 3:** Characteristics of all studies included in the systematic review. Studies are organized by drug, with the name of each drug reported in the first column.

Drug	Author and year	CASP final score	Drug dose (N) *vs* comparator (N)	Length of treatment	Alcohol consumption at inclusion	Additional (psychological) support	Sleep-related outcomes
Disulfiram	Snyder et al., 1981	16	Disulfiram 250 mg (13) *vs* Placebo (17)	7 d	3 w of sobriety before inclusion	None	*Number of REM episodes (mean* ± *SD)* Disulfiram pre 3.69 ± 0.83 post 3.23± 0.88^c^ Placebo pre 3.66 ± 0.77 post 3.56± 1.05^d^ *Latency Stage* 1-REM (mean ± SD) Disulfiram pre 68.81 ± 30.65 post 95.27± 25.44^c^ Placebo pre 59.78 ± 25.86 post 73.09± 35.07^c,d^ *Total REM Time (mean* ± *SD)* Disulfiram pre 101.86 ± 25.18 post 83.13± 23.61 Placebo pre 99.65 ± 32.21 post 92.12± 24.56^a’b^ *% Stage 3* (mean ± SD) Placebo pre 4.49 ± 3.07 post 3.96 ± 3.29^d^ *% Stage 4* (mean ± SD) Disulfiram pre 5.60 ± 7.80 post 8.74 ± 8.02^c^ Placebo pre 7.23 ± 8.58 post 5.37 ± 8.48^d^ a. p < 0.05 pre *vs.* post b. p < 0.05 Disulfiram (difference pre-post) *vs.* Placebo (difference pre-post) c. p < 0.10 pre *vs.* post d. p < 0.10 Disulfiram (difference pre-post) *vs* Placebo (difference pre-post)
						
Acamprosate	Boeijinga et al., 2004	11	Acamprosate 2 g/d (12) *vs* Placebo (12)	23 d	Alcohol allowed (8 d); abstinent 15 d	None	During the 8 d run-in period and the 15 d withdrawal, insomnia was experienced only by placebo subjects (no number of subjects)
	Namkoong et al., 2003	14	Acamprosate 1.332- 1.998 g/d (72) *vs* Placebo (70)	8 w	No sobriety before inclusion	Out-patient psychosocial intervention	*Sleeping difficulty*: 8.3% Acamprosate 6/72 (no data on placebo) and no difference with placebo
	Perney et al., 2012	18	Acamprosate 2-3 g/d (335) *vs* Placebo (257)	24 w	> 10 d	None	*Sleep Disorders Symptoms (SDS)* - Baseline Placebo 40.2% *vs.* Acamprosate 40.7% *SDS-free patients after 6 M* Placebo 34.95% *vs.* Acamprosate 52.21% p = 0.007
	Staner et al., 2006	14	Acamprosate 0.666 g/d (12) *vs.* Placebo (12)	23 d	Alcohol intake allowed for the first 8 d then abstinent for 15 d	None	Day 2 (Withdrawal) - Day 14 (treatment) *Sleep onset latency* (min: mean ±SD) Placebo 25.2 ± 21.3 – 22.7 ± 11.3 Acamprosate 36.6 ± 38.8 – 31.5 ± 20.2 *Total sleep time* (min: mean ± SD)^a^* Placebo 414.9 ± 41.3 – 398.6 ± 59.8 Acamprosate 410.7 ± 35.3 – 402 ± 62 *Total time awake* (min: mean ± SD) Placebo 60 ± 45.9 – 84.3 ± 65.6 Acamprosate 64.3 ± 32.9 – 76.9 ± 59.5 *Sleep efficiency* (%: mean ± SD)^a^** Placebo 87.4 ± 9.5 – 82.8 ± 12.9 Acamprosate 86.6 ± 6.9 – 83.9 ± 12.6 *Wake after sleep onset* (%: mean ± SD)^a^*,^b^* Placebo 8.7 ± 6.6 –13.9 ± 13 Acamprosate 7.9 ± 4.6 –11.5 ±12.1 *Stage 1* (%: mean ± SD) Placebo 4.9 ± 2.4– 4.5 ± 2.1
														Acamprosate 5.3 ± 1.9 – 6.2 ± 2.5 *Stage 4* (%: mean ± SD) Placebo 8.9 ± 6.1– 8.8 ± 6.8 Acamprosate 10.5 ± 6.8 – 9.5 ± 6.3 *REM sleep* (%: mean ± SD) Placebo 22.4 ± 4.2 – 21.2 ± 5.7 Acamprosate 20.3 ± 5.4 – 18.9 ± 6.4 *REM sleep latency* (min: mean ± SD) ^b^* Placebo 66.7 ± 20.3 – 62.6 ± 34.9 Acamprosate 72 ± 18.8 – 82.1 ± 23.5 a) time effect; b) treatment effect *p < 0.05; **p < 0.01
Acamprosate + Naltrexone	Anton et al., 2006	18	No CBI: Naltrexone 100 mg/d (154) *vs.* Acamprosate 3 g/d (152) *vs.* Naltrexone + Acamprosate (148) *vs.* Placebo (153) CBI: Naltrexone (155) *vs.* Acamprosate (151) *vs.* Naltrexone + Acamprosate (157) *vs.* Placebo (156) CBI only (157)	16 w	4 d	CBI only. All 8 groups with medications had 9-session intervention focused on enhancing medication adherence and abstinence	*Somnolence* Placebo 24% Acamprosate 31%^b^ Naltrexone 37%^c^ Acamprosate + Naltrexone 31%^a^ a. p < 0.05 *vs* placebo b. p < 0.01 *vs* placebo c. p < 0.001 *vs* placebo
	Kiefer et al., 2004	9	Naltrexone 50 mg/d (40) *vs.* Acamprosate 1.998 g/d (40) *vs* Naltrexone + Acamprosate (40) *vs.* Placebo (40)	12 w	12-15 d	Coping skills/relapse prevention group therapy	*Sleep Disturbance* (ns) Placebo 11.4% Acamprosate 11.5% Naltrexone 6.3% Acamprosate + Naltrexone 11.5%
Naltrexone	Ahmadi and Ahmadi, 2002	11	Naltrexone 50 mg/d (58) *vs.* Placebo (58)	12 w	3-30 d of sobriety before inclusion		*Lethargy* Naltrexone 22.4% *vs.* Placebo 8.6% *Nightmares/hallucinations* Naltrexone 8.6% *vs.* Placebo 0% *Insomnia* Naltrexone 17% *vs.* Placebo 6.9%
	Ahmadi et al., 2004	11	Naltrexone 50 mg/d (58) *vs.* Placebo (58)	36 w	3-30 d of sobriety before inclusion	Training in relapse prevention (12 weekly of 30 min sessions of individual counselling sessions	*Lethargy* (p = 0.04) Naltrexone 22.4% *vs.* Placebo 8.6% *Nightmares/hallucinations* (p = 0.02) Naltrexone 8.6% *vs.* Placebo 0% *Insomnia* (p = 0.09) Naltrexone 17% *vs.* Placebo 6.9%
	Anton et al., 1999	16	Naltrexone 50 mg/d (68) *vs.* Placebo (63)	12 w	>5 d of sobriety before inclusion	12 weekly sessions of individual manual-guided cognitive behavioral therapy	*Daytime sleepiness* (p < 0.05)Naltrexone 46% *vs* Placebo 27%
	Baltieri et al., 2008	16	Naltrexone 50 mg/d (49) *vs.* Topiramate 300 mg/d (52) *vs.* Placebo (54)	12 w	1 w	Relapse prevention, counseling and encouragement to participate in AA groups	Placebo *vs.* Naltrexone *vs* Topiramate *Insomnia:* 5.6% *vs* 10.2% *v.s* 9.6% ns *Somnolence:* 13.0% *vs.* 20.4% *vs.* 13.5% ns
	Gastpar et al., 2002	16	Naltrexone 50 mg/d (84) *vs.* Placebo (87)	12	Up to 28 d	1 h of psychosodal treatment every wat least	*Somnolence* (ns) Placebo 4% *vs.* Naltrexone 6% *Insomnia (ns)* Placebo 5% *vs* Naltrexone 4%
	Heinala et al., 2001	16	Naltrexone 50 mg/d (63) *vs.* Placebo (58)	12 w + 20 w as needed	1 w	Cognitive coping skills or supportive therapy	*Insomnia* (ns) Naltrexone 3.2% *vs.* Placebo 5.2%
	Johnson et al., 2004	17	Naltrexone IM 400 mg (25) *vs.* Placebo (5)	4 m	> 5 d	Psychosocial treatment	*Somnolence* Naltrexone 12% *vs.* Placebo 0%
Naltrexone	Kranzler et al., 2000	14	Naltrexone 50 mg/d (61) *vs.* Nefazodone 200-400 mg/d (59) *vs.* Placebo (63)	12 w	3-28 d	Weekly coping skills training	*Insomnia* (ns) Placebo 39.7% Nefazodone 35.6% Naltrexone 59.0% *Sleepiness* (ns) Placebo 38.1% Nefazodone 47.5% Naltrexone 47.5%
	Kranzler et al., 2003	12	Naltrexone 50 mg/d (35) *vs.* Naltrexone 50 mg *as needed* (43) *vs.* Placebo/d (39) *vs.* Placebo *as needed* (36)	8 w	Not specified	Biweekly counseling sessions	Difficulty *sleeping* for 17% of the study patients; ns
	Krystal et al., 2001	16	Naltrexone 50 mg/d 12m (209) *vs.* Naltrexone 50 mg/d 3 m- Placebo 9m (209) *vs.* Placebo 12 m (209)	12 m	> 5 d	12-step facilitation counseling (13 m)	*Somnolence* (ns) Naltrexone 5% *vs.* Placebo 2%
	Oslin et al., 1997	16	Naltrexone 50 mg/d (21) *vs.* Placebo (23)	12 w (Monday, Wednesday and Friday)	>6 w of sobriety before inclusion	Psychosocial treatment	*Sleep disturbance* (average duration in w) ns Naltrexone 35% (2.9 w) Placebo 38% (1.4 w)
	Oslin et al., 2008	14	Naltrexone 100 mg/d + CBT (40) *vs.* Placebo+ CBT (40) Naltrexone + BRENDA (39) *vs.* Placebo + BRENDA (40) Naltrexone + doctor (41) *vs.* Placebo + doctor (40)	24 w	3 d of sobriety before inclusion	Psychosocial intervention 1. Cognitive-Behavioral Therapy (CBT) 2. BRENDA 3. Doctor: limited therapeutic content	*Insomnia* (p < 0.01) Naltrexone 24.2% *vs.* Placebo 11.7%
	O’Malley et al., 2008	16	Naltrexone 50 mg/d (34) *vs.* Placebo (34)	16 w	4–30 d of abstinence	9 sessions of medical management and supportive advice	*Sleepiness* (p < 0.001) Placebo 26% *vs.* Naltrexone 35%
	Miranda et al., 2014	13	Naltrexone 50 mg (10) *vs.* Placebo (12)	8-10 d	No sobriety before inclusion	None	*Difficulty sleeping* (ns) Naltrexone 19% *vs.* Placebo 4.8%
Nalmefene	Gual et al., 2013	17	Nalmefene 18 mg (358) *vs.* Placebo (360) *as needed*	24 w	Max 14 d	BRENDA	*Insomnia* Nalmefene two times higher than placebo (no data available)
Acamprosate 5.4 ± 4.9 – 4.8 ± 2.8 *Stage 2* (%: mean ± SD) Placebo 50.9 ± 5.4 – 49 ± 9.6 Acamprosate 52.7 ± 8.4 – 50.2 ± 10 *Stage 3* (%: mean ± SD) ^b^* Placebo 4.9 ± 2.9 – 4.4 ± 2.2
	Karhuvaara et al., 2007	17	Nalmefene 10-40 mg (242) *vs.* Placebo (161) *as needed*	28 w	Max 14 d	Psychosocial intervention (minimal)	*Insomnia* Nalmefene 31% *vs.* Placebo 14% Between group difference (95% Cl) 17 (9.0-25)
	Mason et al., 1999	15	Nalmefene 20 mg/d (35); 80 mg/d (35) *vs.* Placebo (35)	12 w	12 w	Cognitive behavioral therapy weekly	*Insomnia* Placebo 5.7% *vs.* Nalmefene 14.3% (ns)
	Mason et al., 1994	16	Nalmefene 10 mg/d (7) *vs.* Nalmefene 40 mg/d (7) *vs.* Placebo (7)	12 w	No sobriety before inclusion	None	*Insomnia* Nalmefene 36%
	Mann et al., 2013	18	Nalmefene 18 mg (306) *vs.* Placebo (298) *as-needed*	24 w	No sobriety before inclusion	BRENDA	*Sleep disorde*r Placebo 0.3% *vs.* Nalmefene 10.6% *Insomnia* Placebo 3.4% *vs.* Nalmefene 9.9%

AA, alcoholics anonymous; BRENDA, biopsychosocial evaluation, report to the patient on assessment, empathic understanding of the patient’s situation, needs collaboratively identified by the patient and treatment provider, direct advice to the patient on how to meet those needs, assess reaction of the patient to advice, and adjust as necessary for best care; CBI, cognitive behavioral intervention; d, days; w, weeks; m, months; ns, non-significant; IM, intramuscular; SD, standard deviation.

Five studies were pooled in a meta-analysis (Anton et al., 1999; Kranzler et al., 2000; Ahmadi and Ahmadi, 2002; Gastpar et al., 2002; Baltieri et al., 2008): a total of 645 patients with AUD diagnosed with DSM-III-R alcohol dependence (APA. American Psychiatric Association, 1987) were included. The mean age was 42.2 ± 9.0 for naltrexone and 42.8 ± 9.1 for placebo; most patients were males: 76% (naltrexone) *vs.* 72% (placebo), and two studies included only males (Ahmadi and Ahmadi, 2002; Baltieri et al., 2008). One study was based in Iran (Ahmadi and Ahmadi, 2002), two studies in the US (Anton et al., 1999; Kranzler et al., 2000), one in Brazil (Baltieri et al., 2008), and one in Germany (Gastpar et al., 2002; multicentric). The treatment was naltrexone 50 mg/os/day and lasted for 12 weeks. All studies had placebo as comparator; in addition, Baltieri et al. (2008) additionally compared with topiramate and Kranzler et al. (2000) compared with nefazodone.

### Risk of Bias

A total of 26 RCT studies included in the qualitative synthesis were appraised with the CASP checklist for RCT (CASP UK, 2019), and the final scores (out of 18 points) are reported in **Table 3**. Only one RCT was on disulfiram (Snyder et al., 1981) and was of good quality (CASP: 16). Four studies were on acamprosate only, and apart from Boeijinga et al. (2004; CASP: 11), they were all of relatively good quality (CASP: 14–18; Namkoong et al., 2003; Staner et al., 2006; Perney et al., 2012); two RCT studies included both acamprosate and naltrexone, one of good quality (CASP: 18; Anton et al., 2006) and one of lower quality (CASP: 9; Kiefer et al., 2004). The majority of RCT (14 studies) were on naltrexone, and most of them were of good quality (CASP: 14–17; Oslin et al., 1997; Anton et al., 1999; Kranzler et al., 2000; Heinala et al., 2001; Krystal et al., 2001; Gastpar et al., 2002; Johnson et al., 2004; Baltieri et al., 2008; Oslin et al., 2008; O’Malley et al., 2008) while a few were of moderate quality (CASP: 11–13; Ahmadi and Ahmadi, 2002; Kranzler et al., 2003; Ahmadi et al., 2004; Miranda et al., 2014). Finally, five RCT studies were on nalmefene, and they were all of good quality (CASP: 15–18; Mason et al., 1994; Mason et al., 1999; Karhuvaara et al., 2007; Gual et al., 2013; Mann et al., 2013).

The risk of selection bias was mainly unclear, as many studies failed to describe the randomization procedure, while only few studies did (Namkoong et al., 2003; Anton et al., 2006; Karhuvaara et al., 2007; Baltieri et al., 2008; O’Malley et al., 2008; Perney et al., 2012; Mann et al., 2013), and almost none of the studies described the allocation concealment, except for Anton et al. (2006), Gual et al. (2013), Karhuvaara et al. (2007), and Mann et al. (2013).

**Figure 5:**
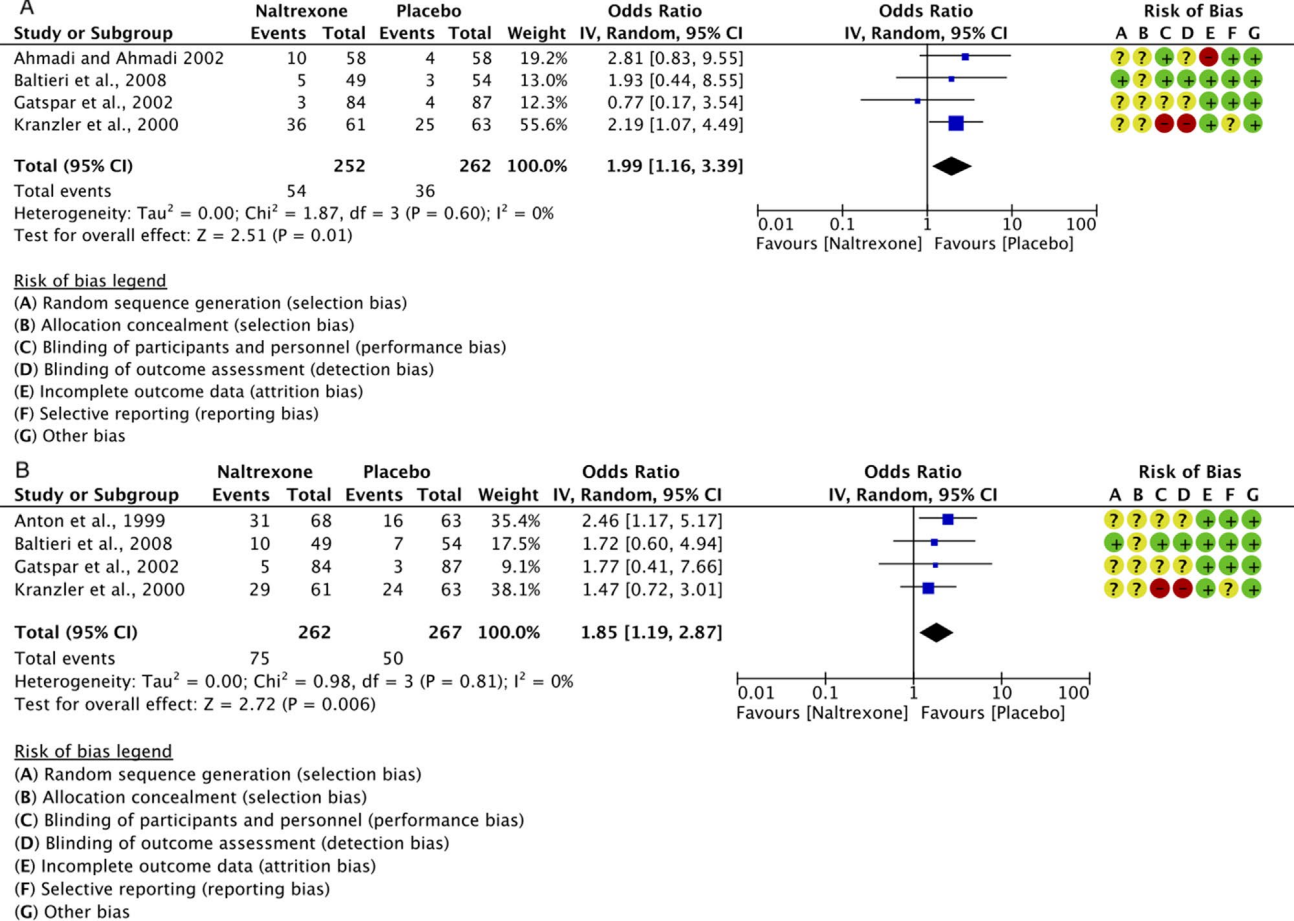
Forest plots of the overall effect of 12-week treatment with naltrexone (50 mg/os/d) or placebo on: insomnia (panel **A**) and somnolence (panel **B**). Each study is shown by a blue square that represents the odds ratio, with the horizontal lines being the 95% confidence intervals; the pooled Odds Ratio and 95% confidence interval by random-effect calculations are depicted as a diamond. Risks of bias are also presented for each study, on the right-up corner.

The risk of performance and detection bias was also unclear for many studies, as only some described the blinding of patients and staff (Snyder et al., 1981; Mason et al., 1999; Ahmadi and Ahmadi, 2002; Namkoong et al., 2003; Ahmadi et al., 2004; Johnson et al., 2004; Anton et al., 2006; Baltieri et al., 2008; O’Malley et al., 2008; Perney et al., 2012; Gual et al., 2013; Mann et al., 2013), while the others described the blinding of patients only, or did not describe any blinding at all; all studies cited a double-blind protocol, except for Kranzler et al. (2000) which was clearly single-blind, with high risk of performance and detection bias. Finally, most studies had low risk of attrition bias, except for Ahmadi et al. (2004), Ahmadi and Ahmadi (2002), Boeijinga et al. (2004); Johnson et al. (2004); Kranzler et al. (2003); Miranda et al. (2014), and Oslin et al. (2008), and low risk of reporting bias, except for Kiefer et al. (2004), Kranzler et al. (2000); Kranzler et al. (2003); Snyder et al. (1981), and Staner et al. (2006), who did not clearly set pre-specified primary and secondary outcomes. The detailed risks of bias of the studies included in the meta-analysis are also reported in (**Figure 5, A and B**). Of all the trials included in the meta-analysis, almost all disclosed the sponsorship, except for: Ahmadi and Ahmadi (2002) and Anton et al. (1999).

## Outcomes

### Qualitative Synthesis

Only three studies focused on sleep as a main outcome, objectively measured by polysomnography (disulfiram: Snyder et al., 1981; acamprosate: Staner et al., 2006) or observed with subjective self-reported sleep from a previous set of data (Perney et al., 2012). All the other studies reported the sleep problems as adverse effects (subjective report).

The main difficulty in the data synthesis was the heterogeneity of measurements, as different studies measured different sleep outcomes, or the same outcomes described by different terms, or non-specified generic outcomes (for example, sleep disturbance; **Table 2**) so the data synthesis and meta-analyses were limited. Sleep outcomes measured as adverse effects included: insomnia, somnolence, sleep disorders symptoms, sleep disorder, sleep disturbance, lethargy, nightmares, sleepiness (which here was considered as a synonym of somnolence; **Table 2**), and difficulty sleeping (which here was considered synonym of insomnia; **Table 2**).

In light of the well-known sleep-altering effects of alcohol (Brooks and Wallen, 2014), it was necessary to eliminate nearly all articles on the effectiveness of disulfiram, since this drug is obviously associated with concomitant alcohol intake to exert its therapeutic effects. Only one study that avoided concomitant use of alcohol, since it focused on sleep, was finally included (Snyder et al., 1981). Disulfiram overall decreases REM sleep, which is shown by a trend to decreased number of REM episodes (p < 0.10), a significantly decreased total REM time (p < 0.05), and a trend to increased latency to REM stage (p < 0.10), compared with placebo. A trend to decreased stage 3 (p < 0.10) was also observed in the disulfiram group compared with placebo (Snyder et al., 1981).

Acamprosate had mainly no/little effect on sleep, which was reported as adverse effect (except for a significantly higher somnolence in the acamprosate group vs. placebo; Anton et al., 2006; p < 0.01). Interestingly, acamprosate seems to give less insomnia than placebo, although the difference was not significant (Boeijinga et al., 2004). In line with these trends, in the only polysomnography study by Staner et al. (2006), acamprosate improved both sleep continuity and architecture with respect to placebo, as shown by a decreased wake time after sleep onset and increased stage 3 and REM sleep latency (p < 0.05 for all measures). This is a very important finding as it shows that acamprosate can be beneficial to ameliorate sleep parameters typically disturbed by AUD. To further confirm the beneficial effects of acamprosate, Perney et al. (2012) showed that after 6 months of treatment with acamprosate, the number of patients self-reporting sleep disorders (symptoms not specified) was decreased with respect to placebo (p = 0.07).

Naltrexone generally increased insomnia, although the difference with placebo was mostly non-significant, except for one study (Oslin et al., 2008; p < 0.01). The strongest effect reported was the increased somnolence (or sleepiness) in the naltrexone group compared with the placebo, which was significant in three studies (Anton et al., 2006; p < 0.001; Anton et al., 1999; p < 0.05; O’Malley et al., 2008; p < 0.001) but non-significant in another four studies, or the significance was not reported.

Nalmefene gave more insomnia than placebo, although the significance was either null or not reported. Somnolence was not reported.

Overall, other parameters that were reported for most drugs were: lethargy, nightmares/hallucinations, and sleep disorders (not specified), but the differences with placebo were non-significant or the significance was not shown.

### Meta-Analysis

Data from five RCT studies (Anton et al., 1999; Kranzler et al., 2000; Ahmadi and Ahmadi, 2002; Gastpar et al., 2002; Baltieri et al., 2008) on self-reported insomnia or somnolence were grouped together in two meta-analyses performed with the Cochrane’s Review Manager (RevMan, 2014).

Insomnia: for a total of 514 individuals, the calculated OR was 1.99 (95% CI: 1.16–3.39) (**Figure 5A**). This indicates a significantly higher risk of insomnia with naltrexone compared to placebo (p = 0.01). No heterogeneity was detected, with a chi-square value of 1.87 (p = 0.60) and an I^2^ of 0%.

Somnolence (daytime sleepiness, **Table 2**): for a total of 529 individuals, the calculated OR was 1.85 (95% CI: 1.19–2.87) (**Figure 5B**). This indicates a significantly higher risk of somnolence with naltrexone compared to placebo (p = 0.006).

No statistically significant heterogeneity was detected with a chi-square value of 0.98 (p = 0.81) and an I^2^ of 0%.

## Discussion and Future Directions

To date, this is the first systematic review evaluating the sleep-related effects of disulfiram, acamprosate, naltrexone, or nalmefene reviewed together.

As reported by Snyder et al. (1981), disulfiram decreases REM sleep in acute abstinent alcoholics. Interestingly, disulfiram inhibits the production of noradrenaline by blocking the dopamine β-hydroxylase enzyme (Goldstein et al., 1964), and this may underlie the decreased REM sleep. In fact, although noradrenergic neurons show only minimal activity during REM sleep, it is recognized that minimal brain stem level of NA is necessary for the occurrence of REM sleep (Gottesmann, 2008), as REM sleep can be suppressed by lesioning the noradrenergic *nucleus, locus coeruleus* (Jouvet and Delorme, 1965). The disulfiram-induced reduction of REM sleep is quite interesting if we consider that during alcohol withdrawal, the REM sleep is usually increased (Brower, 2001). In addition, other psychiatric disorders often associated with AUD and withdrawal, such as depressive disorders, are characterized by increased REM sleep, while on the contrary, antidepressant drugs reduce REM sleep (see Wichniak et al., 2017). In this framework, it could be tempting to suggest that the reduced REM sleep may play an important role in the therapeutic effects of disulfiram, contributing maybe to mood improvements during abstinence, but unfortunately, there are no additional studies on disulfiram to further corroborate this hypothesis.

Acamprosate shows some beneficial effects on sleep, reducing insomnia in some studies (Boeijinga et al., 2004; statistics not known), in line with a recent meta-analysis reporting lowered insomnia after 6 months of treatment with acamprosate *versus* placebo (Perney and Lehert, 2018). However, in this systematic review, we haven’t included most of the studies that were analyzed in Perney and Lehert (2018) meta-analysis, mainly for lack of sleep outcomes (Pelc et al., 1992; Whitworth et al., 1996; Geerlings et al., 1997; Pelc et al., 1997; Poldrugo, 1997; Chick et al., 2000; Tempesta et al., 2000; Mason et al., 2006). In fact, Perney and Lehert (2018) analyzed the raw sleep data collected from those trials. Moreover, two articles on acamprosate were excluded because they were not in English (Barrias et al., 1997; Ladewig et al., 1993) and another two studies were excluded because there was concomitant administration of other, potentially biasing, drugs (Besson et al., 1998; Chick et al., 2000). Finally, due to the high level of methodological heterogeneity across the included studies, we estimated that it was not appropriate to pool the data in a meta-analysis. But the results summarized in this review suggest beneficial effects of acamprosate (**Table 3**), particularly on parameters of sleep continuity and architecture that are usually affected by AUD, giving less fragmented sleep, increased deep sleep (stage 3), and increased REM sleep latency (Staner et al., 2006). Acamprosate, by reducing the glutamatergic neurotransmission (Mason and Crean, 2007; Frye et al., 2016), supports abstinence, and this might explain the reduced insomnia and the trend to reduce REM sleep (Jones, 2019). Glutamate is the major excitatory neurotransmitter in the brain and plays an essential role in regulating wakefulness and REM sleep (Jones, 2019). Indeed, glutamatergic system dysregulation in AUD is a likely substrate for several sleep disturbances (Koob and Colrain, 2019).

Naltrexone alters the sleep–wake cycle giving increased insomnia and somnolence in some studies, although statistical significance was present only in few studies reported in **Table 3** (Anton et al., 1999; Anton et al., 2006; O’Malley et al., 2008; Oslin et al., 2008). The higher risk of insomnia and somnolence with naltrexone compared to placebo is further confirmed by the results showed in the meta-analyses (**Figures 5A, B**, respectively). Despite the lack of evidence on the effect of naltrexone on sleep architecture, preliminary results from a recent polysomnography study on six healthy males demonstrated that a single dose of naltrexone significantly increased stage 2 sleep and decreased REM sleep (Sramek et al., 2014). The observed effects on REM sleep seem to mimic other drugs such as benzodiazepines, tricyclic antidepressants, selective serotonin reuptake inhibitors, trazodone, and opioids (Kay et al., 1969; Cronin et al., 1995; Obermeyer and Benca, 1996; Gursky and Krahn, 2000; Foldvary-Schaefer et al., 2002; Barbanoj et al., 2005; Shaw et al., 2005), all known to affect the central neurotransmitters involved in the sleep–wake cycle, such as catecholamines, acetylcholine, GABA, and histamine (Jones, 2019).

Interestingly, it must be noted that there is a common signature across these three drugs (disulfiram, acamprosate, and naltrexone) in the alteration of sleep architecture. In fact, from the few polysomnography studies available, it seems that all these drugs decrease the REM sleep (Snyder et al., 1981; Staner et al., 2006; Sramek et al., 2014), similarly to antidepressants (see Wichniak et al., 2017), although with different mechanisms of action.

Finally, similar trends for increased insomnia were observed with nalmefene, although much less evidence was available, and the statistical significance was null or not reported. These results are not surprising, given that both naltrexone and nalmefene act on opioid receptors, although with different mechanisms. Opioids modulate various neurotransmitters including acetylcholine (Illes, 1989; Mulder and Schoffelmeer, 1993), catecholamines (Gunne, 1959; Moore et al., 1965), and GABA (Jalabert et al., 2011; Horsfall and Sprague, 2017), and these neurotransmitters are well known to regulate the sleep–wake cycle (see Jones, 2019). More studies are required to establish the exact mechanisms of the opioid-induced effects on sleep–wake cycle.

A growing body of literature shows frequent association of AUD with sleep disorders (Brower, 2003; Arnedt et al., 2007; Angarita et al., 2016). Given that sleep disturbances could have a crucial role in the risk of relapse (Brower, 2001), an appropriate treatment of sleep problems may support the treatment of AUD. The sedative-hypnotic benzodiazepines have been widely used to treat sleep problems as well as acute alcohol withdrawal (Gensburger and Ghuysen, 2019); however, the use of these drugs is not appropriate for people with AUD, for the risk of dangerous interactions with alcohol, and for their potential to induce dependence (Ashton, 2005). It would be unsafe to prescribe benzodiazepines to a population that is clearly more prone to developing drug dependence and can relapse into concomitant heavy drinking, with the risk of drug-alcohol interaction. Recent alternatives to treat sleep disorders in AUD patients involve the newer GABA agonist, well known as Z-hypnotics or Z-drugs (zolpidem, zopiclone, and zaleplon). These Z-drugs enhance GABA_A_ receptor currents *via* a mechanism that differs from that proposed for benzodiazepines (Dixon et al., 2015) and are indicated for the treatment of insomnia characterized by difficulties with sleep initiation. Although these drugs show lower dependence than benzodiazepines, there is still concern over the non-negligible potential of abuse of the “Z” hypnotics, which would be unsafe in people with history of drug addiction (Hajak et al., 2003), and could also interact with alcohol. Recent clinical trials have demonstrated that suvorexant, a dual hypocretin/orexin receptor, normalizes sleep in patients with primary insomnia. More recent studies suggest its potential for the treatment of sleep pathology associated with AUD (Sanchez-Alavez et al., 2019). Campbell et al. (2018) suggested that suvorexant may also address key physiological components related with alcohol abstinence and withdrawal, such as sleep disorders, which should in turn help decrease or inhibit relapse (Campbell et al., 2018). Although these therapeutic approaches seem promising, there is increasing concern around the concomitant use of too many drugs in healthcare. Patients with AUD are at high risk of pharmacological interactions, due to the incidence of comorbidities, the concomitant intake of numerous drugs, and the pharmacodynamic and pharmacokinetic interferences of alcohol (Guerzoni et al., 2018). On the other hand, the use of hypnotic drugs among people with AUD could contribute to overdose mortality, a serious public health concern (Foulds et al., 2018). The combination of drugs, called polypharmacy, exposes the patients to a dangerous vicious circle, as more drugs means more side effects often requiring additional treatments, thus increasing the risk of drug interactions (Marengoni and Onder, 2015). Moreover, a recent systematic review with meta-analysis reported that the additional pharmacotherapy of insomnia in patients with AUD doesn’t improve alcohol abstinence (Miller et al., 2017). Therefore, it is particularly important to elucidate the effects of the pharmacotherapy for AUD on sleep quality, as this may help tailor the treatment based on the sleep profile of AUD patients. A thorough choice of the appropriate pharmacotherapy for AUD with the lowest negative impact (or the highest positive impact) on sleep may better support the crucial phase of the acute withdrawal from alcohol and result in higher success. In this respect, it appears that acamprosate is the most beneficial (or less detrimental) drug for patients with AUD with history of sleep disturbances. However, as highlighted by this review, there is no sufficiently detailed data to corroborate this. In addition, this systematic review has important limitations, such as the inclusion of limited number of articles that are sometimes outdated, especially on disulfiram (as this is a relatively old drug). Most importantly, as sleep measures were almost always reported as adverse effects, it was not possible to determine at what point in the study these were assessed and how was the sleep quality before the treatment, and this represents a potential bias.

In conclusion, given the high burden that sleep disturbances pose on AUD patients, further polysomnography studies to elucidate the effects of these drugs on sleep architecture are long overdue. More robust evidences on the effect of these drugs on sleep architecture may suggest a more holistic approach to treat AUD that takes into consideration sleep as a crucial element of the therapy, with benefits for the patients and maybe higher success.

## Author Contributions

FP designed the protocol and screened articles for eligibility criteria based on titles and abstracts. FP and AP independently screened the full texts of the pre-selected studies and agreed on final inclusion/exclusion of each study. Any disagreements were resolved through consensus. FP and AP drafted the manuscript, figures and tables, and provided editorial supervision. Both authors discussed the results and reviewed the manuscript.

## Conflict of Interest

The authors declare that the research was conducted in the absence of any commercial or financial relationships that could be construed as a potential conflict of interest.
